# Pathogenesis of Abdominal Aortic Aneurysms: Role of Nicotine and Nicotinic Acetylcholine Receptors

**DOI:** 10.1155/2012/103120

**Published:** 2012-03-18

**Authors:** Zong-Zhuang Li, Qiu-Yan Dai

**Affiliations:** Department of Cardiovascular Medicine, First People's Hospital, Shanghai Jiao-Tong University School of Medicine (SJTUSM), Shanghai 200080, China

## Abstract

Inflammation, proteolysis, smooth muscle cell apoptosis, and angiogenesis have been implicated in the pathogenesis of abdominal aortic aneurysms (AAAs), although the well-defined initiating mechanism is not fully understood. Matrix metalloproteinases (MMPs) such as MMP-2 and -9 and other proteinases degrading elastin and extracellular matrix are the critical pathogenesis of AAAs. Among the risk factors of AAAs, cigarette smoking is an irrefutable one. Cigarette smoke is practically involved in various aspects of the AAA pathogenesis. Nicotine, a major alkaloid in tobacco leaves and a primary component in cigarette smoke, can stimulate the MMPs expression by vascular SMCs, endothelial cells, and inflammatory cells in vascular wall and induce angiogenesis in the aneurysmal tissues. However, for the inflammatory and apoptotic processes in the pathogenesis of AAAs, nicotine seems to be moving in just the opposite direction. Additionally, the effects of nicotine are probably dose dependent or associated with the exposure duration and may be partly exerted by its receptors—nicotinic acetylcholine receptors (nAChRs). In this paper, we will mainly discuss the pathogenesis of AAAs involving inflammation, proteolysis, smooth muscle cell apoptosis and angiogenesis, and the roles of nicotine and nAChRs.

## 1. Introduction

Abdominal aortic aneurysms (AAAs) usually occur naturally in the infrarenal part in the human abdominal aorta. In men aged 65–80 years, the prevalence of AAAs is between 4% and 8% and approximately six times greater in men than women [1, 2]. An AAA is a permanent localized dilatation of the abdominal aorta (beginning at the level of the diaphragm and extending to its bifurcation into the left and right common iliac arteries in human) that exceeds the normal diameter by 50%, or >3 cm [3].

The primary risk factors of AAAs include family history, smoking, increasing age, male gender, central obesity, and low HDL-cholesterol levels [2, 4]. Hypertension (systolic BP > 160 mmHg, diastolic BP > 95 mmHg) is associated with the AAA risk, but only in women [5]. Diabetes, a well-defined risk factor for atherosclerosis, has been shown to be protective against the AAAs [6–8].

Historically, the AAAs have been considered as a focal manifestation of the advanced atherosclerosis [9]. However, this conventional theory has been challenged by recent evidences: an AAA was a local representation of a systemic disease of the vasculature [10]. There was a lower incidence of AAAs in the individuals suffering from diabetes mellitus that ordinarily considered as the risk equivalent of atherosclerosis [6–8]. The inflammatory cells were recruited into the different sites: the outer media and adventitia of aneurysma, and the intima and subendothelium of atheroma [11–13].

Three key processes contribute to the AAA phenotype: inflammation, proteolysis, and smooth muscle cell (SMC) apoptosis [2]. On the basis of the loss of extracellular matrix especially elastin and accumulation of proteolytic enzymes in the aneurysmal tissues, proteolysis has been regarded as the critical pathogenesis of AAAs [14]. The extracellular matrix degradation by both predominant proteolytic enzymes MMP-2 and -9, which synthesized and released mainly by the vascular SMCs and infiltrating inflammatory cells such as macrophages, contribute to the anoikis of vascular SMCs. The vascular SMC apoptosis is another critical pathogenesis of AAAs. It has been demonstrated that the decreasing number of the medial vascular SMCs in the vascular wall from the AAAs patients was relevant to apoptosis [15–21]. Degradation of elastin and apoptotic cell death of the medial vascular SMCs destroys the aortic wall integrality, weaken the wall tensile strength, consequently facilitate the development of AAAs. The inflammatory responses in vascular wall play a pivotal role in the MMPs expression and vascular SMC apoptosis. Conversely, the apoptosis and antigen exposure as a result of the extracellular matrix degradation are also likely to contribute to the immune and/or inflammatory responses. Therefore, the initiating factors of AAAs remain mysterious. The mechanism underlying the inflammatory responses in the outer media and adventitia of the vascular wall remains to be well defined. Recent researches have shown that the increased angiogenesis in all layers of the aneurysmal wall is associated with inflammatory responses and related to aneurysmal rupture [22–25].

Cigarette smoking is the irrefutable risk factor of AAAs. It has recently been demonstrated by Stolle et al. [26] that cigarette mainstream smoke enhanced the AAA formation in Ang II-treated apolipoprotein E-deficient mice as a result of the increased proteolytic activity of MMPs. Nicotine, a major alkaloid in tobacco leaves and a primary component in cigarette smoke, plays its pathophysiological roles partly through its receptor—nicotinic acetylcholine receptors (nAChRs). In this paper, we will mainly discuss the pathogenesis of AAAs involving inflammation, proteolysis, vascular SMC apoptosis and angiogenesis, and the roles of nicotine and nAChRs.We made the highlighted change according to the list of references. 

## 2. Nicotine and nAChRs

Nicotine is a principal tobacco alkaloid occurring to the extent of about 1.5% by weight in commercial cigarette tobacco and comprising about 95% of the total alkaloid content. The nicotine in tobacco is largely the levorotary (*S*)-nicotine, only 0.1 to 0.6% of total nicotine content is dextro-rotatory (*R*)-nicotine [27].

There are two major types of cholinergic receptors: the muscarinic and the nicotinic. The endogenous ligand, acetylcholine stimulates both receptor types, while the exogenous one, nicotine, preferentially stimulates nAChRs. The nAChRs were firstly identified in excitable cells, but later were identified in many other cell types including vascular and immune/inflammatory cells. There are 17 distinct isoforms (*α*1–*α*10, *β*1–*β*4, *δ*, *γ*, and *ε*) of the subunits, which form homomeric or heteromeric channels. Among the subtypes, the “muscle-type” nAChR*α*1, the five polypeptide subunits (*α*1, *β*1, *δ*, and *ε* in a 2 : 1 : 1 : 1 ratio), and the homomeric “CNS-type”, *α*7-nAChRs, have been identified in a variety of non-neuromuscular cell types such as vascular ECs, vascular SMCs, smooth muscle specific *α*-actin positive myofibroblasts, T lymphocytes, and macrophages [28–32]. It has been shown that nAChRs, particularly “muscle-type” nAChRs *α*1 and homomeric CNS-type *α*7 nAChRs had participated in the pathological processes of atherosclerosis and angiogenesis [33].

## 3. Nicotine, nAChRs, and Inflammation in AAAs

Inflammation plays a pivotal role in the formation and progression of AAAs and aneurysm rupture [34, 35]. The inflammatory cells including T, B lymphocytes, neutrophils, macrophages, and MCs mostly are recruited into the outer media and adventitia of the aneurysmal wall [13, 22, 24, 36, 37]. Periaortic adipose tissue may also be one of the resident sites of inflammatory cells. Police et al. [38] have demonstrated that the increased number of macrophages in periaortic adipose tissue surrounding the abdominal aortas of Ang II-infused obese mice was associated with the enhanced AAA formation. The inflammatory cells release not only photolytic enzymes to degrade elastin and other matrix proteins, but also inflammatory and chemotactic factors to recruit more inflammatory cells and stimulate the vascular SMC synthetic phenotype by autocrine/paracrine. In previous studies, macrophages have been frequently examined and shown its indispensability in AAAs. T lymphocytes are not indispensable in the AAAs induced by Ang II in apolipoprotein E-deficient male mice, although which play a dominant role in atherosclerosis [39, 40]. Recently, the role of MCs in the AAA development has also been paid more attention by scientists. The specific granule contents from MCs are very important for the inflammatory cell recruitment, pro-MMP and renin-angiotensin system activation, angiogenesis, and vascular SMC apoptosis [36, 41].

It has been shown by few studies that nicotine played a proinflammatory role in vasculature *in vivo* and *in vitro*. Two *in vitro* experiments have demonstrated that nicotine promoted the VCAM-1 and ICAM-1 expression on human coronary artery endothelial cells and human umbilical vein endothelial cells [42, 43]. In another study, chronic (during 90 days) nicotine exposure enhanced the production of pro-inflammatory cytokines such as TNF*α*, Interleukin 1*β* (IL-1*β*) by macrophages and upregulates the mRNA expression level of VCAM-1, cyclooxygenase-2 (COX-2), and platelet-derived growth factor *β* (PDGF-*β*) in the aortas from low-density lipoprotein receptor-deficient mice [44]. It has been well known that VCAM-1 and ICAM-1 were the key mediators of the inflammatory cell migration and infiltration into vascular wall [45]. Nevertheless, more evidences have demonstrated the anti-inflammatory role of nicotine via nAChRs, that is, the so-called cholinergic anti-inflammatory pathway [46–48]. If a hypothesis that “nicotine can stimulate formation and progression of AAAs through inflammation” is true, is it the best explanation that the prolonged exposure to nicotine may induce desensitization and changes in the expression of nAChRs and thus the beneficial effects of nicotine through its receptors may be halted? [49, 50]. It must be conceded that the AAAs were usually detected in the older people with a longer smoking history [49].

Moreover, it has been indicated by some investigations that the inflammatory mediators including COX-2 and 5-lipoxygenase (5-LO) were also associated with the development of AAAs.

COX-2, a limiting enzyme converting arachidonic acidinto prostaglandin, plays an important role in the inflammatory diseases. In human AAAs, the increased expression of COX-2 is associated with the augmented angiogenesis [51]. King et al. demonstrated the increased expression of COX-2 and the upregulated synthesis of PGE_2_ selectively in the aortic aneurismal tissues by exposure to Ang II. The selective COX-2 inhibitor, celecoxib, decreased the incidence and severity of Ang II-induced AAAs in apolipoprotein E-deficient mice and C57BL/6J mice [52]. The above studies indicate that the increased COX-2 expression is one of the pathogenesis of AAA formation. It has been implied by limited studies that nicotine could stimulate the COX-2 expression likely through nAChRs. In human umbilical vein endothelial cells, nicotine increases the COX-2, ICAM-1, and PGE_2_ expression through NF-*kappa*B activation which mediated by nAChRs [53]. In gastric cancer, nicotine stimulates the COX-2 expression to trigger tumor cell invasion and angiogenesis through the VEGF activation, which subsequently modulates the MMP activity and plasminogen activators expression [54].

Activation of the 5-LO pathway contributes to the biosynthesis of proinflammatory leukotriene mediators in macrophages, MCs, and other inflammatory cells [55]. 5-LO plays a role in promoting the AAA formation induced by an atherosclerotic diet in apolipoprotein E-deficient mice. 5-LO-positive macrophages localize in the adventitia of the diseased mouse and human arteries in the areas of neovascularization and constitute a major component of the aortic aneurysms. 5-LO deficiency attenuates the aortic aneurysms and reduces the aortic MMP-2 activity and diminished plasma macrophage inflammatory protein-1*α* (MIP-1*α*) [56]. It has been recently shown that the mRNA levels for the three key enzymes/proteins in the biosynthesis of cysteinyl-leukotrienes, 5-LO, 5-LO-activating protein (FLAP), and LTC_4_ synthase (LTC_4_S), were significantly increased in the aneurysmal wall from the human abdominal aortas. 5-LO, FLAP, and LTC_4_S proteins express in the media and adventitia and localize in the areas rich in inflammatory cells including macrophages, neutrophils, and MCs. Exogenous LTD_4_ increased the MMP-2 and -9 release [57]. Houard et al. have similarly demonstrated that, in the aneurysmal wall of the human abdominal aortas, the leukotriene pathway mainly localized in the macrophage-rich adventitial areas [58]. It has been recently indicated that nicotine could induce the 5-LO expression in colon neoplasm [59]. A hypothesis: “smoking promotes pathogenesis of aortic aneurysm through the 5-lipoxygenase pathway.” Had been proposed by Takagi and Umemoto [60] in 2005, but to date, it remains to be demonstrated.

Taken together, it has been demonstrated by compelling evidences that the inflammation in vascular wall is one of the pathogenesis of AAAs. Mediated by the inflammatory cells such as macrophages and MCs and inflammatory mediators including VCAM-1, ICAM-1, COX-2, and 5-LO, the inflammatory responses have a preference for the outer media and adventitia of the aneurysmal wall. Currently, the notion that nicotine promotes the AAA formation by its receptor nAChRs is still not supported by robust evidences. Fortunately, in our recent animal experiment, the AAAs have been successfully induced by both nicotine and Ang II in the older C57BL/6J mice, accompanied with the MC degranulation in the adventitia of the abdominal aortas. Maybe, it will point out a direction for further research.

## 4. Nicotine, nAChRs, and Proteolysis Induced by MMPs in AAAs

Although the abundant connective tissue proteinases including MMPs (MMP-1, -2, -3, -9, -12, and -13), serine proteases (tissue-type plasminogen activator (t-PA); urokinase-type plasminogen activator (u-PA); plasmin; and neutrophil elastase), as well as cysteine proteases (cathepsin D, K, L, and S) [61] have been described in the human AAA tissues, the most attentions have been kept on the members of the matrix metalloproteinase family [24, 62–65]. Previous studies have focused on the 92-*kD* (MMP-9; gelatinase B) and 72-*kD* (MMP-2; gelatinase A) gelatinase/type IV collagenase, both most prominent elastolytic enzymes secreted by the AAA tissues in organ culture and *in vivo, *which are expressed by macrophages, vascular SMCs, fibroblasts, or ECs, most often in the areas adjacent to the infiltrated inflammatory cells [62, 66–70]. Therefore, it has been shown a close relationship between MMPs and inflammatory responses in the aneurysmal tissues.

The MMPs are a group of zinc-mediated enzymes present in the extracellular matrix. It is a fundamental pathogenesis of AAAs that the increased MMPs in vascular wall degrade all kinds of extracellular matrix proteins, particularly elastin [14, 71]. The MMPs are inhibited by the specific endogenous TIMPs, which comprise a family of four protease inhibitors: TIMP-1, -2, -3 and -4. An imbalance in the proteolytic equilibrium between MMPs and TIMPs is a significant factor of the AAA formation [72]. Elastin and collagens type I/III keep the integrality and elasticity of vascular wall, and resist stretch. Under normal conditions, the content of the proteins keeps balance between degradation and synthesis. But in fact, the balance is principally maintained by the collagen metabolism, because elastin is synthesized and deposited in the early childhood and no further significant synthesis occurs in adult life [73]. The content of collagens type I/III increases *compensatively in *the early stage of the disease, while decreases dramatically in the advanced stage. Degradation of elastin and loss of collagens during the advanced stage destroy the wall integrality and weaken the wall tensile strength, which promotes the development and rupture of AAAs. It is supposed that the degradation of elastin is likely to exert a more significant role in the initiating process of AAAs.

It has been recently demonstrated that cigarette mainstream smoke could enhance the proteolytic activity of MMPs including MMP-2 and -9 induced by Ang II and accelerate both formation and severity of AAAs in the hypertensive apolipoprotein E-deficient mice, [26] while cigarette smoke extract significantly downregulated TIMP-3 in aortic endothelial cells [74]. Similarly, nicotine increases the MMPs (especially MMP-2 and -9) expression and activities in the vascular wall components including ECs, vascular SMCs and infiltrating inflammatory cells such as neutrophils and macrophages, [54, 75–80] and decreases the expression of TIMP-1, -3, and -4 in osteoblasts [81]. Moreover, the endogenic ligand of nicotine, **α*7*-nAChRs, is also involved in the MMP-2 and -9 upregulation in human retinal microvascular endothelial cells [80].

Taken together, nicotine and/or its ligantd **α*7*-nAChRs have been involved in the synthesis and release of proteolytic ingredients MMP-2 and -9 and decreased the TIMPs expression *in vivo* and *in vitro*, thus very likely to be involved in the pathogenesis of AAAs.

## 5. Nicotine, nAChRs, and Medial Vascular SMC Apoptosis in AAAs

Histological examinations of both animal and human experimental AAAs have revealed a paucity of medial vascular SMCs in these specimens which are associated with the SMC apoptosis [15–21]. Vascular SMCs synthesize and release the extracellular matrix proteins including collagens, elastin, glycoproteins, and proteoglycans to provide the mechanical integrality to the vascular wall. In the aortic media, collagens are primarily synthesized by vascular SMCs. Theoretically, a paucity of medial vascular SMCs caused by apoptotic cell death, can reduce the synthesis of extracellular matrix proteins and collagens turnover, eventually attenuate the mechanical properties of the aortic wall and result in the formation and complications of AAAs. Henderson et al. have demonstrated that vascular SMC apoptosis, macrophages, and T lymphocytes coexisted in the aortic mediaand companied by the upregulation of proapoptotic initiators, such as Fas/FasL in the human AAA segments obtained from the patients undergoing open repair [15]. Recently, Yamanouchi et al. [82] firstly reported the direct links between medial vascular SMC apoptosis and pathogenesis of AAAs. In an Ang II-induced aneurysm model in apolipoprotein E-deficient mice, a novel caspase inhibitor Q-VD-OPh, inhibited apoptosis by blocking activation of caspases, drastically reduced the number of infiltrating macrophages and CD3^+^ T cells and remarkably decreased the interleukin-6 level as well as the elastase activity. These findings suggested that inhibition of apoptosis may attenuate the aneurysm formation not only by preventing the vascular SMC depletion but also by affecting the vascular inflammation and matrix degradation.

In *in vitro* studies, cigarette smoke extract causes apoptosis of aortic SMCs, human umbilical vein endothelial cells, pulmonary artery endothelial cells, and aortic endothelial cells from human and rodent animals [83–86]. Beyond our expectations, as a major ingredient of cigarette smoke, nicotine inhibits apoptosis of aortic SMCs and ECs through nAChRs in several investigations [87, 88]. Anyway, the viewpoint that nicotine is involved in the formation and progression of AAAs through apoptotic mechanism seems to be not supported by existing evidences.

## 6. Nicotine Stimulates Angiogenesis through ***α***7-nAChRs in AAAs

Angiogenesis is the new blood vessel formation from pre-existing blood vessels and is a prominent feature in both atherosclerosis and AAAs [23, 25, 89]. In 1996, Thompson et al. [23] had demonstrated that the density of the newly formed vessels was increased in all layers of the aneurysmal wall compared with the control samples. The degree of neovascularization is correlated with the extent of the inflammatory infiltration. Angiogenesis is associated with the inflammatory responses in the aneurysmal wall and accelerate the aneurysm rupture [22–25].

Usually, angiogenic stimuli (e.g., hypoxia or inflammatory cytokines) may induce the expression and release of angiogenic growth factors such as vascular endothelial growth factor (VEGF) and fibroblast growth factor (FGF). These growth factors stimulate the proliferation of ECs in the existing vasculature and migration through the tissue to form new endothelialized channels [90, 91]. Almost all subunits of nAChRs are expressed on ECs, whereas the most abundant receptor subunit is **α*7*-nAChRs [92]. Accumulated evidences have shown that, through excitable *α*7-nAChRs on the plasma membrane of vascular SMCs or ECs, nicotine could stimulate the proliferation and migration of ECs, increase the VEGF and FGF release by vascular SMCs and ECs and promote angiogenesis [29, 54, 79, 93–96]. Moreover, the VEGF receptor and *α*7-nAChRs appear to mediate the distinct but interdependent pathways of angiogenesis [97].

Neovascularization is not the exclusive characteristic of AAAs, which also exists in the atherosclerotic lesion, chronic ischemia, tumor and other illnesses. Therefore, angiogenesis may not be a causative but a “contributing to progression and rupture of aneurysm” factor in the pathological process of AAAs.

It has been recently implied by a chronic nicotine exposure experiment in an hindlimb ischemic mice model that chronic nicotine exposure impaired the angiogenic response to ischemia mediated partly by downregulation of the vascular **α*7*-nAChRs, as well as by a reduction in plasma VEGF level [98]. However, because the researches did not assess the functional measures of limb perfusion, the conclusion remains to be further confirmed.

## 7. Conclusion

It is irrefutable that cigarette smoking is the principal risk factor for AAAs, but as a primary ingredient of cigarette smoke, nicotine, which has role in AAAs is undefined. The increased MMPs expression and degradation of all kinds of extracellular matrix proteins, particularly elastin, in the aneurysmal wall, has been demonstrated by compelling evidences, and nicotine may be involved in the process. The angiogenesis stimulated by nicotine may be the important factor for the progression or rupture of AAAs rather than the causative factor. Inflammation and apoptosis of vascular SMCs, two important pathogenesis of AAAs, are seemingly irrelevant to nicotine. Whether nicotine is the key component in cigarette smoke promoting the formation and progression of AAAs remains equivocal. In a chronic nicotine exposure experiment during 90 days, the inflammatory responses in the aorta are enhanced [44]. It may be explained as the desensitization and changes in the expression of nAChRs and thus the beneficial effects of nicotine through its receptors may be halted [49, 50]. Moreover, the adverse events of nicotine can be attributed to its dose-dependent effects, with the toxic cardiovascular effects at higher doses [49, 93, 99]. Today, cigarette smoking remains a serious social problem. Nicotine replacement therapy is often used in smoking cessation, although there are little evidences on the safety and efficacy of long-term use. Therefore, it is absolutely necessary to clarify the exact roles of nicotine in AAAs, especially the adverse effects of chronic exposure.

## Abbreviations

AAA: Abdominal aortic aneurysm
MMP: Matrix metalloproteinase
TIMP: Tissue inhibitors of metalloproteinase
HDL: High density lipoprotein
nAChRs: Nicotinic acetylcholine receptors
SMC: Smooth muscle cell
EC: Endothelial cell
MC: Mast cell
CNS: Central nervous system
AngII: Angiotensin II
VCAM-1: Vascular cellular adhesion molecule-1
ICAM-1: Intercellular adhesion molecule-1
COX-2: Cyclooxygenase-2
5-LO: 5-lipoxygenase
FLAP: 5-LO-activating protein
PGE_2_: Prostaglandin E_2_
MIP-1*α*: Macrophage inflammatory protein-1*α*
LTC_4_S: Leukotriene C_4_ synthase
LTD_4_: Leukotriene D_4_
VEGF: Vascular endothelial growth factor
FGF: Fibroblast growth factor.
